# Development and validation of the Vanderbilt PRS-KS, an instrument to quantify polygenic risk score knowledge

**DOI:** 10.1016/j.gimo.2023.100822

**Published:** 2023-06-01

**Authors:** Doug Stubbs, Gillian W. Hooker, Yajing Li, Lucas Richter, Alexander Bick

**Affiliations:** 1 Pathology, Microbiology, and Immunology, Vanderbilt University Medical Center, Nashville, TN; 2 Concert Genetics, Nashville, TN; 3 Division of Genetic Medicine, Department of Medicine, Vanderbilt University Medical Center, Nashville, TN

**Keywords:** Polygenic risk score, Genetic knowledge, Genomic risk score, Knowledge scale, Scale validation

## Abstract

**Purpose:**

As polygenic risk scores (PRSs) enter clinical practice, health care providers’ and the publics’ comprehension of PRS results are of great importance; yet, they are poorly understood. We present the Vanderbilt polygenic risk scores knowledge scale (Vanderbilt PRS-KS), a tool to quantify PRS knowledge.

**Methods:**

The Vanderbilt PRS-KS was developed by a team of genetic counselors and physicians to cover key conceptual facts pertaining to PRSs. We recruited (*n* = 500) individuals with demographics representative of a U.S. sample and graduate-level health care students (*n* = 74) at a large academic medical center to participate in this validation study. We evaluated the Vanderbilt PRS-KS’s psychometric properties using confirmatory factor analysis and item response theory.

**Results:**

The 7-item Vanderbilt PRS-KS correlated to a single latent construct on confirmatory factor analysis (Λ = 0.31-0.61). The scale showed promising reliability (Cronbach’s α = 0.66) with item response theory summed scores of ≥2 to ≤5, demonstrating reliability >0.70. The Vanderbilt PRS-KS significantly correlated with genetic knowledge and applied PRS knowledge (r = 0.55 and r = 0.29), and graduate-level health care students scored significantly higher compared with the U.S. representative sample (*P* < .01).

**Conclusion:**

The Vanderbilt PRS-KS is a rigorously validated measure to quantify PRS knowledge. Clinicians should tailor future PRS knowledge interventions to the identified knowledge gaps, including PRS inheritance, equity of performance in different ethnicities, and integration with other health determinants.

## Introduction

Complex disorders are caused by alterations in many genes with small effect sizes, often coinciding with environmental factors, which influence an individual’s risk of developing disease over time.^1^ The genetic contributions of many complex diseases are still being examined. Genome-wide association studies elucidate variant associations in many genes to outcomes of interest, which researchers quantify and aggregate to form polygenic risk scores (PRSs).^2^ A PRS is a sum of the established risk alleles, weighted by effect size, on an outcome. PRSs can stratify an individual’s disease risk in combination with traditional risk factors (ie, family history, age, or smoking) or as a potential standalone tool when monogenic and traditional risk factors are insufficient or poorly understood.

As PRSs enter clinical practice, health care providers’ and patients’ comprehension of results and the proper risk communication strategies are of great importance; yet, they are poorly understood. A validated PRS knowledge measure could identify current gaps in knowledge, promote responsible PRS use, and serve as an objective tool for future research. We present the Vanderbilt polygenic risk scores knowledge scale (Vanderbilt PRS-KS), a tool to quantify PRS knowledge. Our goal was to develop and validate a measure with the following criteria: (1) encompass benefits and limitations of PRSs, (2) utilize context-neutral language, and (3) demonstrate sound psychometric properties. To our knowledge, no existing PRS knowledge measure fulfills these criteria.

## Materials and Methods

### Respondents and procedures

The Vanderbilt PRS-KS’s psychometric properties were validated in 500 individuals recruited through prolific.co, an online research and survey distribution platform.^3^ The 500 individuals were representative of U.S. Census Bureau data for age, sex, and ethnicity. Additionally, 74 graduate students at a large academic medical center were recruited to assess convergent validity. Graduate-level health care students were ascertained through recruitment fliers and word of mouth. Study data were collected and managed using REDCap electronic data capture tool.^4^^,^^5^

### Survey measures

#### The applied PRS knowledge measure

The applied PRS knowledge measure is not a published measure and was derived from commercial lab reports. The 3-item applied PRS knowledge measure assesses the individual’s ability to interpret a graphical PRS result and the individual’s understanding of the limitations of the results.

#### Demographics

Demographics were self-reported and included sex, race, and ethnicity.

#### Genetic knowledge

The previously validated 19-item UNC_GKS was used to assess individuals’ genetic knowledge.^6^ The UNC_GKS genetic knowledge includes statements covering genes, genes and health, and how genes are inherited.

#### Attitudes toward genetics

Attitudes toward genetics were measured using the 6-item “favorable attitude” subscale of the genetic attitudes scale by Morren et al.^7^

### Data analysis

Descriptive statistics of the Vanderbilt PRS-KS were reported in the U.S. representative and graduate-level health care students’ samples. The U.S. representative sample was used for the scale’s psychometric analysis, which included internal consistency (quantifies the relationship between a set of items), confirmatory factor analysis (CFA) (confirms a single underlying construct in the scale), item response theory (IRT) (investigates the relationships between responses to each item and the individual’s ability), and convergent validity (evaluates the expected associations with conceptually related variables).

### Item-level descriptive statistics

We found the proportion of individuals correctly answering each item of the Vanderbilt PRS-KS to determine if there were floor effects (<90% answering an item correctly) or ceiling effects (>90% answering an item correctly), indicating whether items are too hard or too easy. Inter-item tetrachoric and item-total correlations verified positive correlations between items before beginning CFA.^8^ Receiving a negative or low item-total or inter-item correlation or violating floor or ceiling effects could indicate that items are not measuring the same latent construct, and items may need to be removed or altered.

### CFA

Using R package lavaan,^9^ 1-factor CFA was performed using the maximum likelihood estimator.^10^ Following suggested reporting guidelines,^11^ acceptable model fit was defined as χ^2^ with a *P* value > .05, Tucker-Lewis index (TLI) and comparative fit index >0.95, root mean square error of approximation <0.05, standardized root mean square residual <0.8, and residual values modification indices <10. Modification indices were used to detect areas of local dependence, and items displaying local dependence were considered for removal.^12^

Standardized factor loadings provide evidence of good item fit to a single underlying construct. Acceptable standardized factor loadings were defined as items with loadings >0.30. Items with loadings <0.30 were removed. CFA was then repeated with the remaining items.^13^

### IRT

Three IRT models were tested for good fit. The 1-, 2-, or 3-parameter logistic IRT model (1PL, 2PL, or 3PL, respectively). The −2∗log-likelihood of 2 model calibrations, for example, 1PL versus 2PL, were used to determine model fit with the null hypothesis that the more parsimonious model fits well.^14^ Item fit was assessed using the Orlando and Thissen’s S-χ^2^ statistic with a (*P* > .05) representing good fit.^15^

IRT follows 4 key assumptions, scale unidimensionality, locally independent items, monotonicity, and item invariance.^14^ CFA confirmed the scale’s unidimensionality. Monotonicity (the assumption that as ability increases, the probability of a correct response increases) was confirmed by the s-shaped curves on the item characteristic curves. Modification indices <10 provided evidence for no local dependencies. Item invariance was examined through differential item functioning (DIF).^16^

DIF was examined using age, sex, race, and ethnicity variables in the U.S. representative sample. Logistic regression models were used to look for a relationship between item responses and subgroups. The first model contained 1 predictor, IRT scores. The second model contained the IRT score predictor and a group membership predictor. The third model included the IRT score predictor, group membership predictor, and their interaction. Uniform DIF was evaluated using a likelihood ratio test comparing model 1 and model 2 and nonuniform DIF by comparing models 2 and 3. After multiple comparisons, the Benjamini-Hochberg procedure with a false discovery rate of 0.05 was applied to determine the presence of significant DIF.^17^

Similar to previous scale development methods, items that displayed CFA or IRT misfit, violated local dependence, or exhibited DIF were removed.^6^ The remaining items were used to calculate the IRT test information function (TIF) and reliability.

### IRT scoring and reliability

The IRT TIF curve was generated using the sum of the item’s information. Higher information represents higher precision at different levels of PRS knowledge.^18^ Typically, IRT scores have a mean of 0 with a standard deviation of 1. To enhance interpretability, summed IRT scores were scaled to the T-score metric, making the mean 50 with a standard deviation of 10.^6^ Each item response was weighted by its discrimination parameter (*a*), with greater discrimination having a more significant impact on the IRT scores. However, multiple combinations of correct and incorrect item responses can generate a summed score >0, regardless of the scaled scores. A scoring table was developed, converting summed scores to expected scale T-score metrics.

### Classical test theory reliability

Internal consistency of the Vanderbilt PRS-KS was computed using Cronbach’s α and the greatest lower bounds (GLB).^19^^,^^20^ Traditionally, acceptable Cronbach’s α values are ≥0.70 and poor values are <0.60.^21^

### Convergent validity

Pearson correlations were calculated between the Vanderbilt PRS-KS and the UNC-GKS, the applied PRS knowledge measure, and the individuals’ attitudes toward genetics. Positive, significant correlations between individuals’ genetic knowledge, applied knowledge, and attitudes toward genetics and Vanderbilt PRS-KS scores were anticipated.

Multivariate linear regression investigated for significant summed Vanderbilt PRS-KS score differences between the U.S. representative sample, graduate-level health care student sample, age, sex, race, and ethnicity. Prior graduate-level genetic training was only collected in the graduate-level health care student sample; therefore, it was not included in this model.

In a different linear regression model utilizing the graduate-level health care sample, receiving graduate-level genetic education was examined for statistical significance using multivariate linear regression with age, sex, race, and ethnicity as the other independent variables.

## Results

### Development of the Vanderbilt PRS-KS

Developing the Vanderbilt PRS-KS was an iterative process of collecting feedback on the scale's wording, clarity, and comprehensiveness, which involved a team of genetic counselors, geneticists, and medical doctors with considerable clinical genetic experience and education. Utilizing existing genetic knowledge scales,^6^^,^^22^^,^^23^ 10 candidate items were piloted for the Vanderbilt PRS-KS (Supplemental Table 1). The Vanderbilt PRS-KS was designed to represent one latent construct, PRS knowledge. Items addressed misconceptions between PRSs and monogenic tests, which could affect decision making (eg, if you receive a high polygenic risk result, your children will have a high polygenic risk result).

### Item retention

Our analytic plan was to drop items that did not fit the CFA or IRT model, violated local dependence, or violated DIF. Items 1 and 2 were removed from the 10 draft items of the Vanderbilt PRS-KS based on low standardized factor loadings (Λ < 0.3) in the initial CFA. After rerunning CFA with the remaining 8 items, draft items 7 and 3 displayed local dependence with modification indices >10. The wording of item 3 (PRSs are based on genetic changes in more than one gene) and item 7 (a PRS can be measured at birth) did not reveal surface-level dependence. To promote the IRT assumption of local independence, we individually removed each item and examined the CFA model fit.

Removing item 3 and retaining item 7 led to poor CFA model fit (TLI = 0.945) and introduced new local dependence between items 5 and 10 (modification indices = 10.1). Removing item 7 and retaining item 3 displayed good CFA fit with no areas of local dependence. Therefore, we kept item 3 and removed item 7 for analysis, leaving the 7-item Vanderbilt PRS-KS (Table 1).

**Table 1:** 7-item Vanderbilt PRS-KS

1. Polygenic risk scores are based on genetic changes in more than one gene.∗^a^
2. All people who receive a high-risk result on a polygenic risk score for a disease will develop that disease.
3. A polygenic risk score can combine other health determinants beyond genetics.∗
4. Polygenic risk scores have the same accuracy regardless of the disease being testing for.
5. Polygenic risk scores have the same accuracy for all people, regardless of their racial or ethnic background.
6. If you receive a high polygenic risk score result, your children will have a high polygenic risk result.
7. Your polygenic risk score could find a decreased risk for disease.∗

a Items marked with ∗ are true.

### Respondents

#### U.S. representative sample

Five hundred individuals completed the survey using prolific.co’s representative U.S. sample algorithm (Table 2). The percentage of male and female respondents was similar (48.8% and 50.4%), with a majority being white (73.8%) and non-Hispanic (91%). The average age was 45 years old.

**Table 2:** Participant characteristics

Demographics	U.S. Representative Sample (*N* = 500)		Graduate-Level Students (*N* = 74)	
	*N* (%)	Mean (SD)	*N* (%)	Mean (SD)
Age (years)		45 (15.9)		26 (5.38)
Sex				
Male	244 (48.8%)		20 (27%)	
Female	252 (50.4%)		53 (71.6%)	
Prefer not to answer	4 (0.8%)		1 (1.4%)	
Race				
White	369 (73.8%)		60 (81.8%)	
Non-White	129 (25.8%)		12 (16.2%)	
Prefer not to answer	2 (0.4%)		2 (2.7%)	
Ethnicity				
Hispanic	41 (8.2%)		9 (12.2%)	
Non-Hispanic	455 (91%)		65 (87.8%)	
Prefer not to answer	4 (0.8%)		0 (0%)	

#### Graduate-level health care students

Seventy-four graduate-level students at a large academic medical center completed the survey. The majority were female, 71.6%; White, 81.8%; and non-Hispanic, 87.5%. The average age was 26 years old.

### Descriptive statistics

#### U.S. representative sample

**Figure 1:**
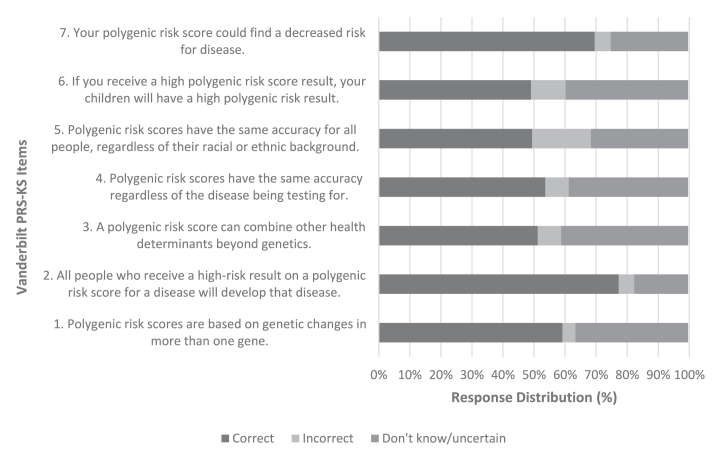
**Item response distribution of the Vanderbilt PRS-KS in the U.S. representative sample.** There was a total of *n* = 500 responses. The mean proportion of individuals correctly answering each question was 0.59 (SD = 0.11).

**Figure 2:**
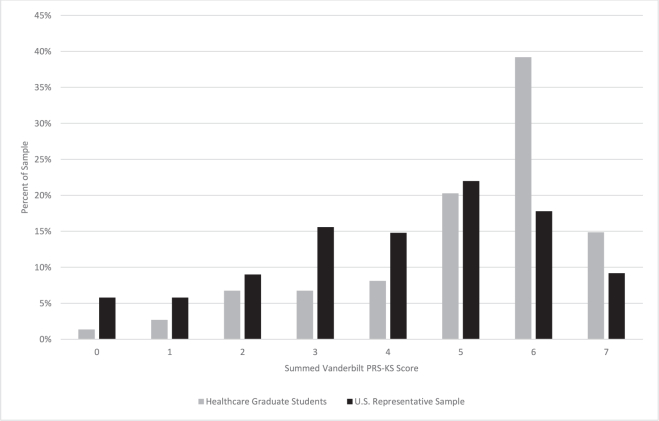
**Summed score distributions of the Vanderbilt PRS-KS in the U.S. representative (*n* = 500) and graduate-level health care students (*n* = 74) samples.** The mean Vanderbilt PRS-KS scores of the U.S. representative and graduate-level health care students were $\bar{x}$ = 4.11 and $\bar{x}$ = 5.09, respectively.

The mean proportion of individuals answering each item correctly was 0.59, with a standard deviation of 0.11 (Figure 1). The proportion of correct responses for each item ranged from 0.49 to 0.78, demonstrating no ceiling or floor effects. The inter-item correlations were positive and moderate to large magnitude r = 0.37, ranging from 0.26 to 0.46. Similarly, the item-total correlations were positive r = 0.59, ranging from 0.49 to 0.78. The mean summed score of the Vanderbilt PRS-KS was $\bar{x}$ = 4.11 with no identified outliers (Figure 2).

#### Graduate-level health care students

**Figure 3:**
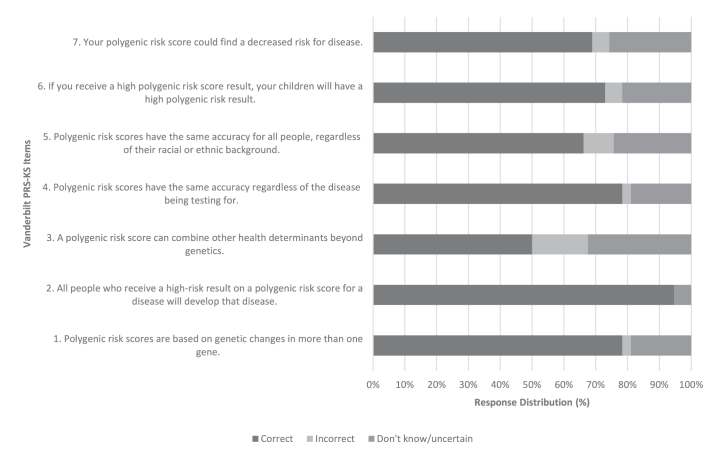
**Item response distribution of the Vanderbilt PRS-KS in the graduate-level health care student sample.** There was a total of *n* = 74 responses. The mean proportion of individuals correctly answering each question was 0.73 (SD = 0.13).

The mean proportion of individuals answering each item correctly was 0.73, with a standard deviation of 0.13 (Figure 3). The mean summed score on the Vanderbilt PRS-KS was $\bar{x}$ = 5.09.

### CFA

The 7-item Vanderbilt PRS-KS displayed acceptable 1-factor CFA fit (χ^2^ = 22.218, df = 14, *P* = .074, root mean square error of approximation = 0.034, comparative fit index = 0.977, and TLI = 0.965). The standardized factor loadings showed moderate item contribution to each factor, Λ = 0.33 to Λ = 0.61, suggesting the 7 items were associated with a single underlying construct, PRS Knowledge (Supplemental Table 2).

### IRT analysis

The Vanderbilt PRS-KS fit the 2PL model better than the 1PL model, meaning that the items’ location (*b*) and discrimination parameter (α) varied (χ^2^ = 35.14, df = 6, and *P* < .01). We compared the 2PL with the 3PL model to determine if individuals were likely to be guessing. The results indicated that individuals were not likely to be guessing and were selecting “don’t know/uncertain” as their response if they were uncertain (χ^2^ < 0.01, df = 1, and *P* = 1.00). The item-level fit was sufficient for the 2PL model (*P* > .05).

The difficulty parameters ranged from *b* = −1.09 to 0.04, indicating modest variation. The discrimination parameters were positive with a moderate slope (*a* = 0.69 to *a* = 1.95), with no evidence of local dependence.

Item characteristic curves followed an s-shaped curve, increasing as ability increased (Supplemental Figure 1). Using a false discovery rate of 0.05, there was no evidence of uniform or nonuniform DIF across age, sex, race, and ethnicity subgroups. We proceeded with the 2PL model for IRT scoring.

### IRT score and reliability

The IRT scores were scaled to an estimated T-score metric for a more straightforward interpretation. Respondents’ IRT T-scores ranged from 29.1 to 65.4 (Supplemental Figure 2). Supplemental Table 3 enables the practical conversion of summed Vanderbilt PRS-KS scores to expected scaled T-scores. The scale’s TIF demonstrated good reliability (≥0.7) for T-scores between 37.28 and 54.76 (Supplemental Figure 3). Converted to summed scores, the Vanderbilt PRS-KS showed good reliability (>0.70) for summed scores ≥2 and ≤5.

### Classical test theory reliability

The Vanderbilt PRS-KS approaches acceptable reliability in the U.S. representative sample with Cronbach’s α = 0.66. The GLB demonstrated acceptable reliability (0.70).

### Convergent validity

Pearson’s correlations demonstrated positive correlations for genetic knowledge and the applied PRS knowledge measure (Supplemental Table 4). The Vanderbilt PRS-KS moderately correlated with the UNC_GKS genetic knowledge measure (r = 0.55) and slightly correlated with the applied PRS knowledge measure (r = 0.29). The respondents’ attitudes toward genetics did not correlate with the Vanderbilt PRS-KS (*P* = .17).

After controlling for age, sex, race, and ethnicity, multivariate linear regression found a significant difference in summed Vanderbilt PRS-KS scores between the graduate-level health care students and the U.S. representative sample (*P* = 1.6e^−5^). Multivariate linear regression found no significant difference in summed Vanderbilt PRS-KS scores between the 2 samples based on age, sex, race, or ethnicity.

Sixteen of the 74 graduate-level health care students received graduate-level genetic education. Receiving graduate-level genetic education did not produce a significant score difference on the Vanderbilt PRS-KS in the graduate-level health care student sample (*P* = .221).

## Discussion

This study addresses several limitations of prior studies exploring patients’ PRS knowledge. First, assessments of PRS knowledge are typically disease specific, usually regarding cancer.^24^, ^25^, ^26^ Although cancer PRSs are the most commonly used clinically, PRSs have shown promising clinical utility for various diseases.^27^ We designed the Vanderbilt PRS-KS to be “context neutral,” ie, not specific to a method of PRS development, disease prediction, or current clinical utility. Therefore, the Vanderbilt PRS-KS’s neutral wording could standardize PRS knowledge measurement in multiple disease settings. Second, existing studies assessing individuals’ PRS knowledge did not report the reliability or validity of their knowledge measures.^24^^,^^25^^,^^28^ Reliability and validity determine how well an instrument measures its intended construct and its consistency across populations and time. Ensuring acceptable reliability and validity of instruments leads to better evidence-based conclusions.

The Vanderbilt PRS-KS was validated using rigorous psychometric methods. IRT has several advantages compared with classical test theory methods, including modeling items individually to the construct measured, allowing for precision estimates across different ability levels of the underlying construct and providing score estimates independent of the items used.^14^ The Vanderbilt PRS-KS is most reliable for low to moderate genetic knowledge (summed scores between ≥2 and ≤5). We expect the general population to fall within 1 standard deviation of the U.S. representative sample mean, in which the Vanderbilt PRS-KS results are most precise. Notably, the Vanderbilt PRS-KS has acceptable reliability (>0.60) for individuals with scores <2, who are more likely to need targeted interventions and support interpreting PRSs.

The summed Vanderbilt PRS-KS scores correlated positively with related concepts, including genetic and applied knowledge, and displayed a significant difference between the U.S. representative and graduate-level health care student sample. We expected the Applied PRS-KS to have the strongest association with Vanderbilt PRS-KS summed scores because it was designed to test applied PRS knowledge. However, we found the UNC-GKS had the highest correlation with summed Vanderbilt PRS-KS scores (r = 0.55). Item 2 on the Applied PRS-KS was more likely to be answered incorrectly than items 1 and 3. This item was the only negatively worded item on the Applied PRS-KS, which respondents could have missed. Also, the answer to item 2 was not included in the Applied PRS-KS graphic making it more difficult to interpret.

No statistical significance was found between summed Vanderbilt PRS-KS scores and attitudes toward genetics or receiving graduate-level genetic education. One possible explanation is the low variation in summed scores on the attitudes toward genetics scale. Most individuals agreed to all items (*n* = 373/500), which can lead to an underestimate of the correlation coefficient (coefficient of variation = 15.62%). Similarly, receiving graduate-level education was not a significant predictor of PRS knowledge in the graduate-level health care student sample. However, only 16 of 74 individuals indicated receiving graduate-level genetic training. The reduced sample size makes it difficult to detect statistically significant results, particularly when the effects are minor.

The Vanderbilt PRS-KS items performed proportionately to similarly worded items on other assessments of PRS knowledge. In the U.S. representative sample, 59% correctly answered item 1 on the Vanderbilt PRS-KS, “Polygenic risk scores are based on genetic changes in more than one gene,” compared with 78% of the graduate-level health care student sample. Similarly, in a cohort of breast cancer patients and family members, 64% correctly answered the true/false statement, “there is more than one DNA change that can increase a woman's risk for breast cancer.”^24^ A study surveying PRS knowledge in health professionals found that 90% of individuals correctly answered “polygenic risk is a cumulative measure of multiple single nucleotide polymorphisms (SNPs).”^25^ The percentage answered correctly was slightly higher in the two comparable cohorts but followed the same trend in our data; higher levels of education that were presumed correlated with a higher proportion of correct responses on related PRS knowledge items.

Interestingly, the U.S. representative slightly outperformed the graduate-level health care sample on items 3 (51%) and 7 (69%) compared with the graduate-level health care student sample (50% and 68%) on the Vanderbilt PRS-KS. Item 3 evaluated knowledge of PRS integration with factors apart from genetics. This is an important concept because PRSs are better predictors in combination with other clinical factors.^27^ Item 3 could have been misinterpreted as the development of PRS can include other health determinants rather than PRSs can be combined with other health determinants. A cohort of (*n* = 223) individuals using an online PRS software found 78.2% correctly answered the item “the results I received show that my lifestyle and environment play no role in whether I develop a health condition.”^28^ There was a large discrepancy in correct responses between the PRS software users cohort and the samples in our validation study. We suspect a disconnect between understanding that environmental and lifestyle factors influence disease and that PRSs can incorporate factors outside of genetics to predict disease, for example, clinical risk factors. Future PRS knowledge interventions should emphasize PRSs’ clinical utility in combination with other existing health predictors.

Item 7 gauged an individual’s knowledge of PRSs reporting decreased risk for disease. It was unclear whether individuals understood some genetic changes in PRSs can be protective against disease. However, when interpreting the Applied PRS Knowledge result, individuals in both cohorts performed better when asked if their PRS results placed them at a higher risk than the general population. These individuals could presumably also interpret a PRS as placing them below population risk. Clinicians should emphasize the possibility of decreased disease risk and provide graphics for relative risk. Incorporating decreased PRS results in the context of other health predictors will be of utmost importance for clinical management.

Items 5 and 6 on the Vanderbilt PRS-KS were the lowest-scored items in the U.S. representative sample, 50% and 49%, respectively. Item 5 measured knowledge of the equity of PRSs across different ethnicities, and item 6 measured the inheritance of PRSs. One barrier to PRS implementation is their limited prediction accuracy in non-European populations.^29^ Recognizing this limitation is important for providers ordering PRSs to interpret them in the context of race and ethnicity. In the previously mentioned study of health professionals,^25^ 82% correctly answered “polygenic risk scores are accurate across all ethnicities.” The health care professionals were mainly genetic counselors with presumable education in genetics and health care disparities and performed significantly higher than this study’s U.S. representative population cohort. The general population deserves similar education on genetic health care disparities. Future interventions should emphasize PRSs are primarily generated from European data and therefore are more accurate in individuals with European ancestry.

Item 6 had the highest proportion of incorrect responses, possibly because of the complicated allele inheritance and weighting not seen in monogenic diseases. Current monogenic educational tools (eg, a pedigree) are ineffective for PRS inheritance education. Previous studies show a proband with at-risk relatives influences the adoption of PRS results.^24^ Therefore, the residual risk for family members should be included in the PRS knowledge interventions. Novel strategies to communicate PRS inheritance would benefit future PRS educational materials.

The present study has several limitations. First, the Vanderbilt PRS-KS’s Cronbach’s α value for reliability is in-between acceptable α ≥ 0.7 and poor values α ≤ 0.6. Cronbach’s α assumptions of tau-equivalence and normality of the data are often unmet and can lead to an under-representation of the true reliability of the scale.^30^ Notably, the GLB value of 0.70 was acceptable in the U.S. representative sample population. Second, our factor loading criteria of ≥0.3 for item inclusion during CFA was relaxed compared with other suggested methods of ≥0.4.^31^ PRS knowledge is a broad construct; therefore, we relaxed the factor loadings to ensure the scale was comprehensive to PRS knowledge. A different factor loading retaining criteria suggested retaining factor loadings that do not cover zero in their 95% CI,^31^ which the Vanderbilt PRS-KS met. Third, for T-scores above 60, the reliability falls below 0.60, indicating that at the extremes of the Vanderbilt-PRS-KS summed test scores, there is less ability to reliably differentiate PRS performance (eg, between individuals who score a 6/7 and a 7/7). However, the graduate-level health care sample was a significant predictor of Vanderbilt PRS-KS performance. This suggests the test can differentiate between individuals based on ability despite the lower reliability in the Vanderbilt PRS-KS summed extremes. Finally, the level of educational attainment in the U.S. representative sample was not collected, which is known to influence genetic knowledge.^32^ In our validation, the graduate-level health care students were presumed to have higher levels of education than the U.S. representative sample. Multivariate linear regression found a significant difference between the Vanderbilt PRS-KS scores in the U.S. representative population and the graduate-level health care student population, suggesting the items demonstrate convergent validity with education level. Future studies should also consider population density as a potential modifier for genetic knowledge.

In summary, we provide the Vanderbilt PRS-KS as a promising tool to quantify PRS knowledge in diverse settings and demographics. The Vanderbilt PRS-KS is comprehensive while retaining brevity. At 7 items, the scale is feasible for clinical and research settings. We highlight specific knowledge gaps for future PRS knowledge interventions, including PRSs’ inheritance, performance in different ethnicities, and integration with other health determinants. Targeted PRS-related knowledge interventions can potentially increase PRS adoption by patients and clinicians, advocate for responsible usage, promote informed decision making, and lead to better outcomes. Adopting the rigorously validated Vanderbilt PRS-KS can standardize the quantification of PRS knowledge and guide future PRS education interventions.

## Data Availability

The data that support the findings of this study are available on request from the corresponding author, D.S.
