# Supplementary material for: Development and validation of the Vanderbilt PRS-KS, an instrument to quantify polygenic risk score knowledge
Source: Genet Med Open. 2023 Jun 1;1(1):100822. doi: 10.1016/j.gimo.2023.100822 (PMC11613715; doi:10.1016/j.gimo.2023.100822)
Supplement: Supplemental Tables and Figures [file mmc1.pdf]

Supplemental Table 1. Vanderbilt PRS-KS Draft Items

1. Finding changes in multiple genes is always a better predictor of disease than finding changes in one gene.
2. Common genetic changes can influence your risk for disease.*[a]
3. Polygenic risk scores are based on genetic changes in more than one gene.*
4. All people who receive a high-risk result on a polygenic risk score for a disease will develop that disease
5. A polygenic risk score can combine other health determinants beyond genetics.*
6. Polygenic risk scores have the same accuracy for all people, regardless of their racial or ethnic background.
7. A polygenic risk score can be measured at birth.*
8. Polygenic risk scores have the same accuracy for all people, regardless of their racial or ethnic background.
9. If you receive a high polygenic risk score result, your children will have a high polygenic risk result.
10. Your polygenic risk score could find a decreased risk for disease.*

[a] items marked with * are True.

Supplemental Table 2. Results of One-Factor Confirmatory Factor Analysis and Two-Parameter Logic Item Response Theory Model for the Vanderbilt PRS-KS

Item Number	CFA[a] Model	2PL IRT[b] Model Parameters				2PL IRT Model Item Fit	
	Λ	*a*	SE[c]	*b*	SE	S- χ^2	df[d]
1	0.33	0.74	0.14	-0.58	0.16	2.71	4
2	0.52	1.65	0.26	-1.09	0.13	8.55	4
3	0.31	0.69	0.14	-0.09	0.15	9.33	4
4	0.61	1.95	0.32	-0.12	0.08	5.00	4
5	0.47	1.22	0.19	0.02	010	2.52	4
6	0.53	1.53	0.23	0.04	0.08	6.13	4
7	0.50	1.39	0.21	-0.81	0.12	1.53	4

[a]CFA = confirmatory factor analysis; [b]2PL IRT = two- parameter logic item response theory; [c]SE = standard error; [d]df = degrees of freedom

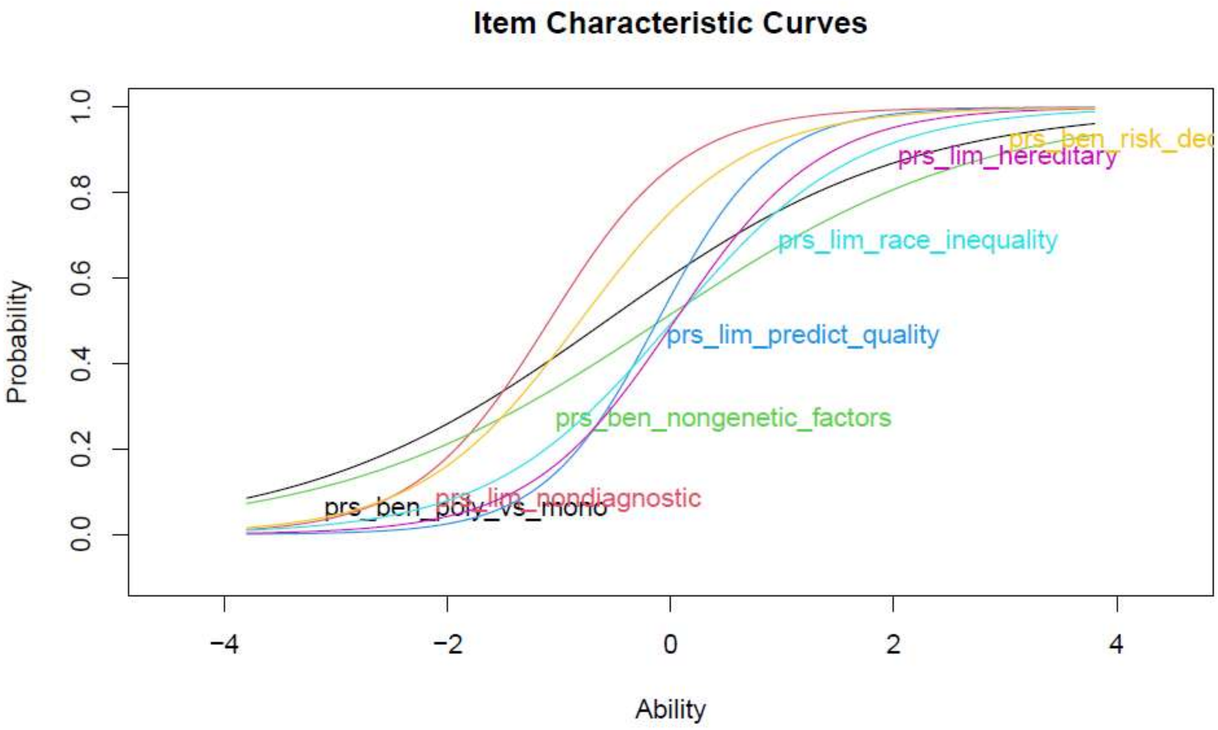

Supplemental Figure 1. Item Response Theory Item Characteristics Curve. Each item on the Vanderbilt PRS-KS has an item characteristic curve labeled with its variable name. The x-axis is the ability level (Θ). Lower ability levels represent lower PRS knowledge levels, and higher ability levels represent higher PRS knowledge. The y-axis indicates the probability of individuals endorsing a question correctly from 0.0 to 1.0. Items shifted to the left on the x-axis are considered easier items (requiring a lower level of PRS knowledge to endorse correctly). In comparison, items to the right are considered more challenging items.

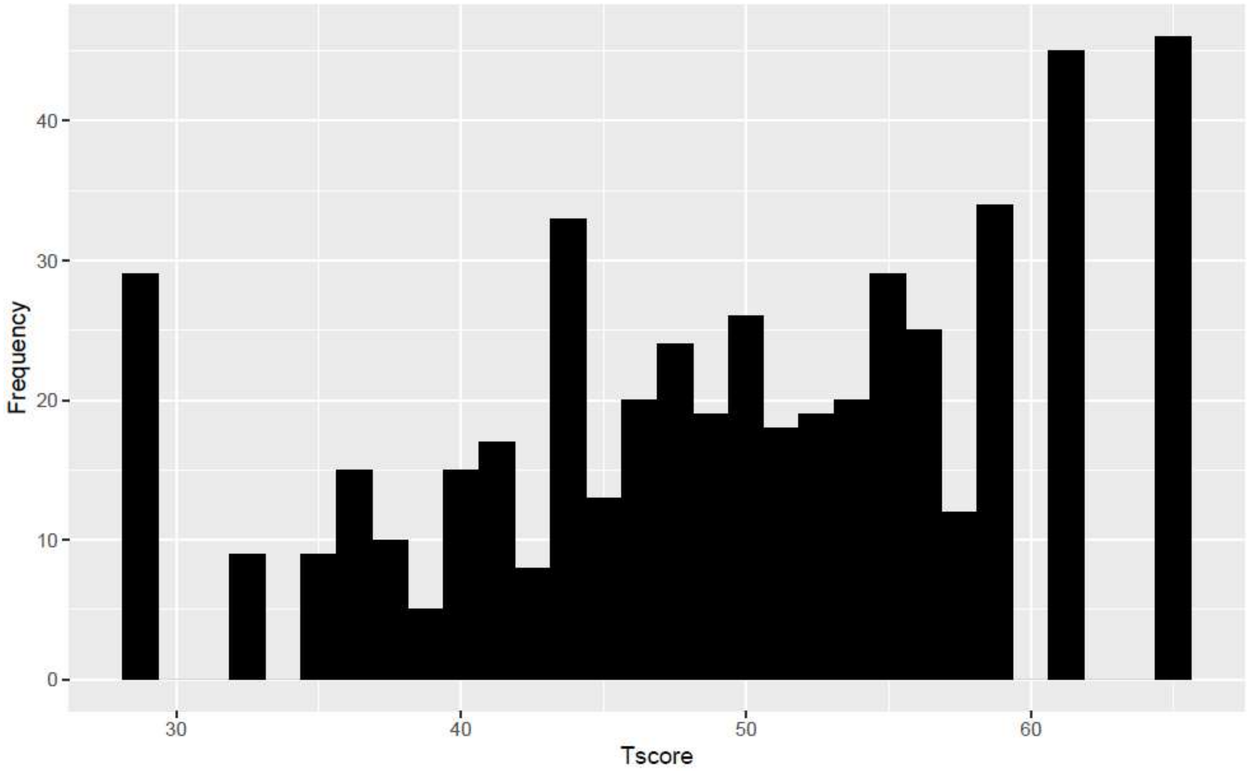

Supplemental Figure 2. Item Response Theory Score Distribution Scaled to T-Scores. The x-axis represents the summed IRT scores scaled to T-scores, with a population mean of 50 and a standard deviation of 10. The y-axis represents the frequency of the scaled T-score.

Supplemental Table 3. Summed Vanderbilt PRS-KS Scores Conversion to Item Response Theory Scaled T-Scores

Summed Score[a]	T-Score	Standard Error
0	29.1	0
1	34.9	1.69
2	39.7	2.16
3	44.2	2.22
4	48.9	2.07
5	54.1	2.22
6	59.9	1.91
7	65.4	0

[a]This table converts the summed count of a participant's correct responses (summed scores) to item response theory T-score metric for comparisons across populations. We calculated the T-scores in the present study's sample using a mean of 50 and a standard deviation of 10. To convert the participant's summed score to the IRT T-Score, find the summed score and move horizontally to the associated T-score.

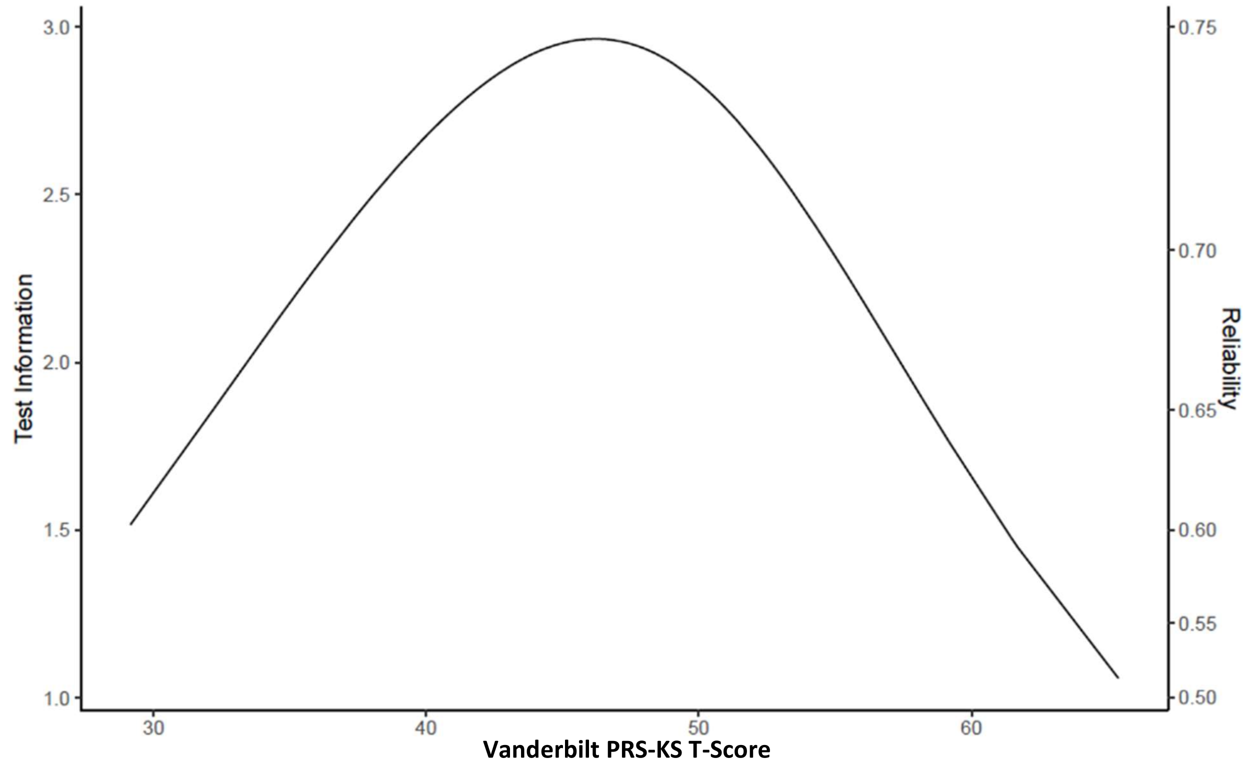

Supplemental Figure 3. Item Response Theory Test Information Curve. The test information curve represents the precision across different levels of PRS Knowledge. The y- axis represents test information, which is the sum of all the items' information. The x-axis is scaled to represent PRS knowledge through T-scores. The T-scores are scaled to a mean of 50 and a standard deviation of 10.

Supplemental Table 4. Pearson Correlations Between Vanderbilt PRS-KS and Additional Measures

Scale	Correlation With Vanderbilt PRS-KS (r)	95% CI
Genetic Knowledge (UNC_GKS)	0.55*[a]	0.48-0.60
Attitudes Towards Genetics	0.06	-0.03-0.15
Applied PRS Measure	0.29*	0.21-0.37

[a] Items marked with * are $P < 0.001$; Note: CI = Confidence Interval
