# Supplementary material for: Development and validation of the Vanderbilt PRS-KS, an instrument to quantify polygenic risk score knowledge
Source: Genet Med Open. 2023 Jun 1;1(1):100822. doi: 10.1016/j.gimo.2023.100822 (PMC11613715; doi:10.1016/j.gimo.2023.100822)
Supplement: Supplemental data [file mmc2.pdf]

Resize font:

# Polygenic Risk Score Knowledge Scale

**Please complete the survey below.**

Thank you!

1) **Please provide your Prolific ID**
* must provide value

## Demographic Information

We are collecting these demographics to look for possible correlations with Polygenic Risk Scores (PRS) knowledge. *Please answer all of the questions before moving on to the next section.*

2) **What is your current age?**
* must provide value

Please express age as an integer.

3) **Sex**
* must provide value

- Male
- Female
- Other/Prefer not to answer

4) **Race**
* must provide value

- White
- Non-White
- Prefer not to answer

5) **Ethnicity**
* must provide value

- Hispanic
- Non-Hispanic
- Prefer not to answer

## Polygenic Risk Scores Knowledge Scale (PRS_KS)

This survey is trying to better understand how people learn about new types of genetic tests, as we recognize people learn in different ways. The following questions are specifically asking about a type of genetic test called **polygenic risk scores**.

Below is a list of statements. Each statement is either true or false. For each statement:
· Select True if you think it is true
· Select False if you think it is false
· Select Don't know/uncertain if you are not sure or don't know

*Please answer all of the questions to the best of your ability.*

6) **Finding changes in multiple genes is always a better predictor of disease than finding changes in one gene.**
* must provide value

- True
- False
- Don't know/uncertain

7) **Common genetic changes can influence your risk for disease.**
* must provide value

- True
- False
- Don't know/uncertain

**8) Polygenic risk scores are based on genetic changes in more than one gene.**

* must provide value

- True
- False
- Don't know/uncertain

**9) All people who receive a high-risk result on a polygenic risk score for a disease will develop that disease.**

* must provide value

- True
- False
- Don't know/uncertain

**10) A polygenic risk score can combine other health determinants beyond genetics.**

* must provide value

- True
- False
- Don't know/uncertain

**11) Polygenic risk scores have the same accuracy regardless of the disease being testing for.**

* must provide value

- True
- False
- Don't know/uncertain

**12) A polygenic risk score can be measured at birth.**

* must provide value

- True
- False
- Don't know/uncertain

**13) Polygenic risk scores have the same accuracy for all people, regardless of their racial or ethnic background.**

* must provide value

- True
- False
- Don't know/uncertain

**14) If you receive a high polygenic risk score result, your children will have a high polygenic risk result.**

* must provide value

- True
- False
- Don't know/uncertain

**15) Your polygenic risk score could find a decreased risk for disease.**

* must provide value

- True
- False
- Don't know/uncertain

## UNC-GKS 19 Items Shortened Version

This survey is attempting to quantify genomic knowledge.

Below is a list of statements. Each statement is either true or false. For each statement:
· Select True if you think it is true
· Select False if you think it is false
· Select Don't know/uncertain if you are not sure or don't know

*Please answer all of the questions to the best of your ability. Don't worry if you do not know the right answers. We do not expect you to answer all of these correctly.*

**16) Genes are made of DNA.**

* must provide value

- True
- False
- Don't know/uncertain

**17) Genes affect health by influencing the proteins our bodies make.**
* must provide value

- True
- False
- Don't know/uncertain

**18) All of a person's genetic information is called his or her genome.**
* must provide value

- True
- False
- Don't know/uncertain

**19) A person's genes change completely every 7 years.**
* must provide value

- True
- False
- Don't know/uncertain

**20) The DNA in a gene is made of four building blocks (A, C, T, and G).**
* must provide value

- True
- False
- Don't know/uncertain

**21) Everyone has about 20,000 to 25,000 genes.**
* must provide value

- True
- False
- Don't know/uncertain

**22) Gene variants can have positive effects, harmful effects, or no effects on health.**
* must provide value

- True
- False
- Don't know/uncertain

**23) Most gene variants will affect a person's health.**
* must provide value

- True
- False
- Don't know/uncertain

**24) Everyone who has a harmful gene variant will eventually have symptoms.**
* must provide value

- True
- False
- Don't know/uncertain

**25) Some gene variants have a large effect in health, while others have a small effect.**
* must provide value

- True
- False
- Don't know/uncertain

**26) Some gene variants decrease the chance of developing a disorder.**
* must provide value

- True
- False
- Don't know/uncertain

**27) Two unrelated people with the same genetic variant will always have the same symptoms.**
* must provide value

- True
- False
- Don't know/uncertain

**28) Genetic disorders are always inherited from a parent.**
* must provide value

- True
- False
- Don't know/uncertain

**29) If only one person in the family has a disorder, it can't be genetic.**
* must provide value

- True
- False
- Don't know/uncertain

**30) Everyone has a chance for having a child with a genetic disorder.**
* must provide value

- True
- False
- Don't know/uncertain

**31) A girl inherits most of her genes from her mother, while a boy inherits most of his genes from his father.**
* must provide value

- True
- False
- Don't know/uncertain

**32) A mother and daughter who look alike are more genetically similar than a mother and daughter who do not look alike.**
* must provide value

- True
- False
- Don't know/uncertain

**33) If a parent has a harmful gene variant, all of his or her children will inherit it.**
* must provide value

- True
- False
- Don't know/uncertain

**34) If one of your parents has a gene variant, your brother or sister may also have it.**
* must provide value

- True
- False
- Don't know/uncertain

## Attitude Towards Genetics

This part of the survey is attempting to judge your attitude towards genetics.

Below is a list of statements related to genetics. For each statement:
· Select Agree if you agree with the statement
· Select Disagree if you disagree with the statement
· Select Don't know if you do not have an opinion for the statement

*Please provide your honest opinions to the following statements.*

**35) I think the development of DNA research is hopeful for the treatment of diseases.**
* must provide value

- Agree
- Disagree
- Don't know

**36) I think the development of DNA research is a positive medical progress.**
* must provide value

- Agree
- Disagree
- Don't know

**37) I approve of using DNA-testing for early detection of diseases.**
* must provide value

- Agree
- Disagree
- Don't know

**38) I would inform my children about the results of a DNA-test for a specific disease.**
* must provide value

- Agree
- Disagree
- Don't know

**39) I want to know whether my disease is hereditary.**
* must provide value

- Agree
- Disagree
- Don't know

**40) I would inform my siblings about the results of a DNA-test for specific disease.**
* must provide value

- Agree
- Disagree
- Don't know

# Applied PRSs Knowledge

This survey is attempting to quantify your applied knowledge of polygenic risk scores. You will be given a sample polygenic risk result. Please use the provided result, visual aid, interpretation, and description to answer the following questions.

Below is a list of statements. They are either true or false. For each statement:·

· Select True if you think it is true
· Select False if you think it is false
· Select Don't know/uncertain if you are not sure or don't know

*Please answer all of the questions to the best of your ability.*

**Please use the report and accompanying text to answer the following questions.**

**Results-** 33.5 % Remaining Lifetime Risk for Breast Cancer
7.0% Five-year Risk for Breast Cancer

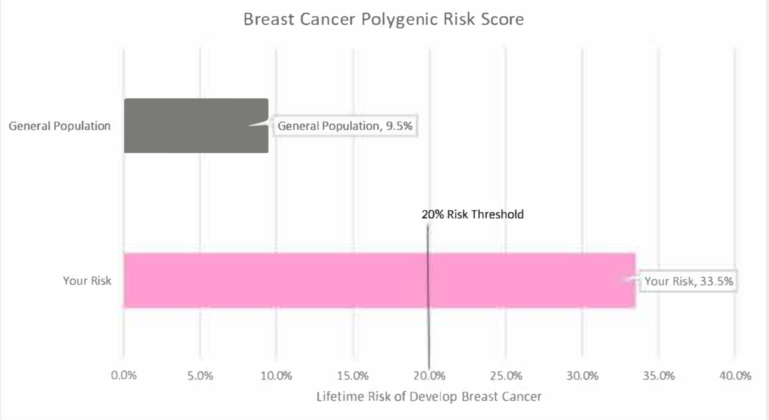


**Breast Cancer Polygenic Risk Score Interpretation**

The breast cancer polygenic risk score provides an estimate of the remaining lifetime risk for breast cancer. A risk estimate at or above 20% has specific medical recommendations, including aggressive breast cancer screening. Women with a risk estimate below 20% may still require medical management based on other clincial factors.

**Breast Cancer Polygenic Risk Score Analysis Description**

The breast cancer polygenic risk score's 5-year and lifetime breast cancer risks is based on an analysis of genetic markers. 27 biomarkers were analyzed using 20% next generation sequencing (NGS). The allele status of these markers is weighted and combined to create the breast cancer polygenic risk score. Missing clinical data and cancer family history may lead to an over- or under-estimate of breast cancer risk.

**41) Your genetic changes placed you above the genetic risk of the general population.**
* must provide value

○ True ○ False ○ Don't know/uncertain

**42) Your lifetime breast cancer risk could not change if additional environmental factors were included.**
* must provide value

○ True ○ False ○ Don't know/uncertain

**43) From your current age, you have a 33.5% lifetime risk of developing breast cancer.**
* must provide value

○ True ○ False ○ Don't know/uncertain

**Submit**
